# Team‐Based Learning in the Meta Horizon Workroom: A Pilot Study on Its Potential Effectiveness for Pharmacology Teaching

**DOI:** 10.1002/prp2.70170

**Published:** 2025-08-25

**Authors:** Abdullah Khaiyam, Lucy Battersby, Ellie Porter, Ana Correia de Oliveira, Soban Sadiq

**Affiliations:** ^1^ Kent and Medway Medical School University of Kent Canterbury UK

**Keywords:** Meta Horizon Workroom, pharmacology, students, team‐based learning

## Abstract

The Meta Horizon Workroom (MHW) provides an immersive environment for team‐based learning (TBL). This study evaluated its effectiveness in teaching pharmacology, emphasizing engagement, collaboration, and learning, aiming to assess the potential of MHW in enhancing the learning experience. A pilot study was conducted with Kent and Medway Medical School (KMMS) Year 4 medical students using Meta Quest 2 virtual reality (VR) headsets. Students participated in a virtual TBL session via MHW after a preparatory phase that included studying assigned materials. The session centered on an application exercise, which forms the core of TBL, and focused on solving a pharmacology‐focused case scenario. Data collection involved 5‐point Likert‐scale and open‐ended questions. Descriptive and thematic analyses were performed to assess levels of engagement, collaboration, and learning of pharmacology concepts. Quantitative data indicated that 5 of the 7 participants found the MHW TBL approach more engaging than traditional TBL methods. Furthermore, 6 of the 7 viewed the platform as a potential game‐changer for pharmacology education due to its interactive features. Qualitative feedback emphasized the benefits of interactive tools, peer‐to‐peer learning, and easy access to resources during discussions. However, some challenges, including technical issues during the session, were reported. The findings demonstrate the transformative potential of MHW for pharmacology education, offering an engaging, collaborative, and innovative learning platform. While technical challenges need addressing, this pilot study underscores the value of integrating VR into medical education. This foundational work highlights the promise of immersive virtual environments in revolutionizing pharmacology teaching.

## Graphical abstract

Students feedback on the Meta Horizon Workroom‐based Team‐Based Learning (TBL) approach in pharmacology highlights enhanced engagement, collaboration, and conceptual understanding, with most students recommending its future use as an innovative teaching method.

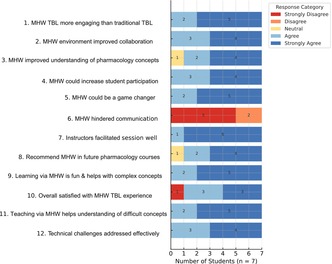

**Abbreviations**
KMMS: Kent and Medway Medical School
MHW: Meta Horizon Workroom
TBL: team‐based learning
VR: virtual reality

## 1 Introduction

The rapid advancement of digital technology has profoundly impacted various fields, including medical education. One of the most promising developments in this regard is the use of virtual reality (VR) platforms to facilitate collaborative learning [1]. The integration of VR into medical education aligns with the growing trend of digital learning tools being used to enhance student engagement and knowledge acquisition [2]. Parong and Mayer [3] demonstrated that immersive technology fosters active participation and reduces cognitive overload by allowing students to interact with 3D models. This insight is particularly relevant in pharmacology, where understanding complex drug interactions and mechanisms is crucial for clinical practice.

The Meta Horizon Workroom (MHW) is a cutting‐edge virtual collaboration space in the metaverse designed to facilitate remote teamwork, discussion, and learning [4]. MHW has the potential to enhance engagement and learning through its interactive and realistic simulations. One of the key advantages of MHW is its ability to foster active learning [5]. This is supported by its design, which promotes direct interaction with content and peers, thereby encouraging deeper cognitive processing. The interactive features of MHW inherently support these principles, fostering environments where students are actively engaged in problem‐solving and critical thinking rather than passive reception [5]. Research has shown that active learning strategies lead to better knowledge retention compared to passive learning approaches [6]. By immersing students in a virtual environment, MHW encourages them to engage with learning materials actively rather than passively absorbing information.

Team‐based learning (TBL) is an active learning and instructional pedagogy that provides students with opportunities to apply conceptual knowledge through a sequence of activities that includes individual work, team work, and immediate feedback [7]. Unlike traditional TBL, in which students interact in a physical classroom, MHW allows learners to engage in interactive discussions, share resources, and collaborate in a virtual space using VR headsets. By integrating visual aids, interactive tools, and a dynamic team‐based structure, MHW has the potential to revolutionize how pharmacology is taught to medical students. Traditional TBL methods faced communication, disengagement, and unequal participation barriers [8]. While traditional TBL can be effective, it sometimes lacks the dynamic and immersive elements that virtual platforms can offer, particularly for distributed teams or where physical interaction is limited. The design of MHW allows for more fluid and integrated interaction, which can address some of the limitations of traditional TBL in fostering a continuously dynamic team environment. Specifically, traditional TBL environments can pose challenges for facilitating equitable participation among all students, especially in larger groups, and may not fully cater to diverse learning styles through varied interactive modalities. MHW seeks to mitigate these issues by providing a highly interactive and engaging virtual space. Being in a setting in which students can experience VR, it can enable them to feel more immersed, engaged, and participative due to its presence and interactive nature [2].

Further research is needed to determine whether VR can increase engagement and collaboration in TBL environments effectively. Although previous studies have shown that VR is useful for medical education [2, 9], the research impact of using MHW in teaching pharmacology remains insufficient. The aim of this initial exploratory study was to investigate how the utilization of TBL in a virtual environment via MHW affected the effectiveness of teaching pharmacology, as perceived by the students. This study aimed to provide insights that may contribute to the growing body of research in this area and highlight the need for further investigation. The objectives were to identify the engagement, collaboration, and learning among participants, including any barriers or challenges as perceived by themselves.

## 2 Methods

Ethical approval for this study was obtained from the KMMS REAG (Research Ethics Advisory Group) (Approval ID: 2405009). A pilot study was conducted with KMMS Year 4 medical students using Meta Quest 2 VR headsets. Students were recruited via an open invitation email sent to the entire Year 4 medical student cohort at KMMS. Recruitment advertisement was also published in student newsletter. Participation was voluntary, and the VR activity was conducted in a dedicated simulation suite during a scheduled non‐curricular session. Inclusion criteria for participants were enrolment as a Year 4 medical student at KMMS, participants comfortable in immersive technology and voluntary consent to participate. Exclusion criteria included any self‐reported history of severe motion sickness or neurological conditions that might be exacerbated by VR use. The TBL session lasted for approximately 60 min, comprising a 10‐min introduction and VR headset training, a 40‐min application exercise and discussion, and a 10‐min post‐session questionnaire. Faculty members (instructors) were present within the MHW session to facilitate discussions and provide immediate feedback. The application exercise primarily involved teamwork, where students collaborated to solve a case scenario; however, initial reading of assigned materials was an individual preparatory task. Students participated in a virtual TBL session via MHW after a preparatory phase that included studying assigned materials. The session centred on an application exercise, which forms the core of the TBL approach, and focused on discussing questions around a pharmacology‐focused case scenario. All students had prior on‐campus TBL experience and sufficient pharmacology knowledge from their first 3 years of study. The assessment of “sufficient pharmacology knowledge” was based on successful completion of prior pharmacology modules in their curriculum, ensuring participants had foundational understanding relevant to the case scenario which revolves around hypertension and its treatment. This was a pragmatic approach for a pilot study to investigate the intervention's effects in a controllable manner, acknowledging that future, larger studies would explore broader applicability.

**FIGURE 1:**
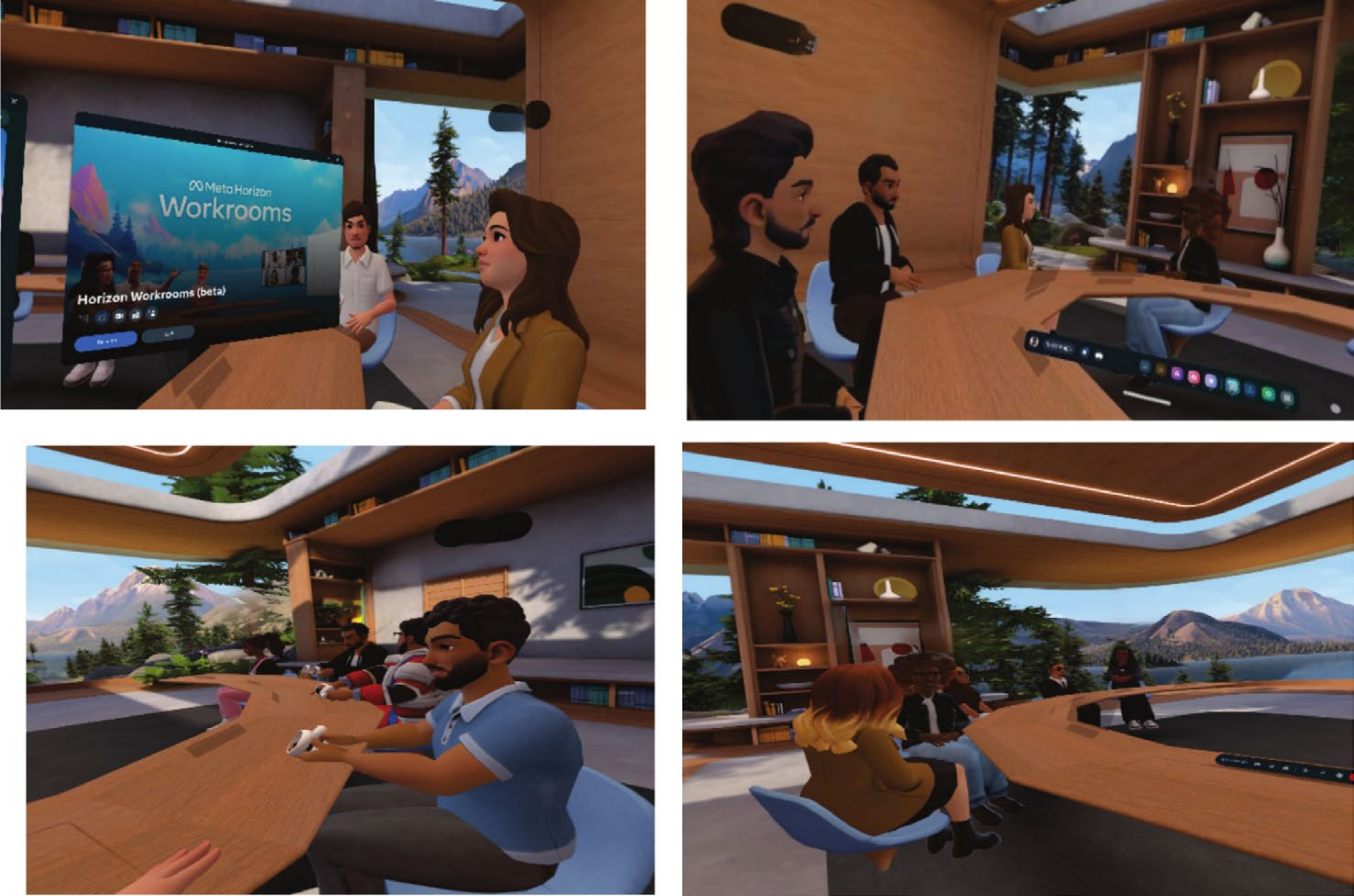
Students participation in Team‐based learning (TBL) via Meta Horizon Workroom (MHW). Once students entered inside the MHW, they created their own Avatars as shown above and started a discussion on the pharmacology clinical case scenario. The figure shows students‐created Avatars and discussion among each other in the MHW environment. No identity of student is revealed in the avatar.

On the day of research, all students completed a cybersickness questionnaire as it has been published that use of VR could cause cybersickness symptoms, for example headache, distress, or general discomfort [10]. Afterwards, a brief MHW and VR headsets training was provided to all students. They then immersed themselves in MHW along with the faculty members. There they created their Avatar (Figure 1) and engaged in the TBL application exercise. On completion, all students and faculty members left MHW and completed the cybersickness questionnaire and a separate questionnaire related to their experience in MHW.

**FIGURE 2:**
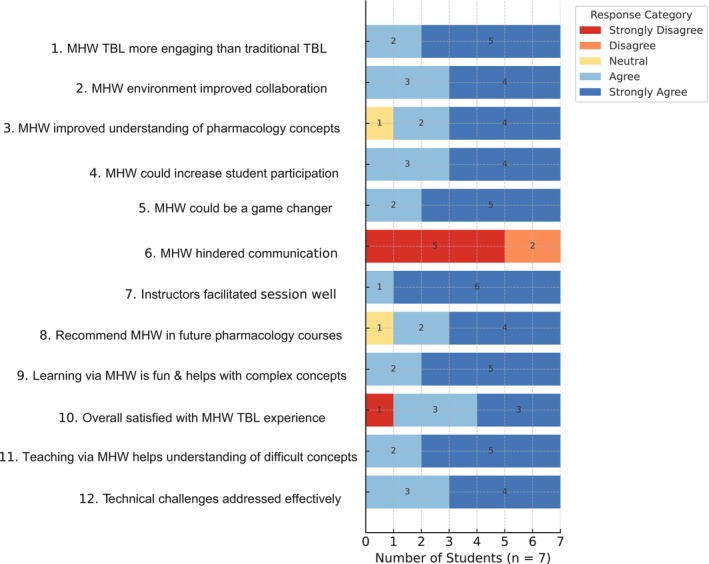
Questionnaire responses (from 7 students) on the effectiveness of Meta Horizon Workrooms (MHW) in pharmacology team‐based learning (TBL). The figure presents the distribution of student responses across a 5‐point Likert scale for various questions (Strongly disagree to Strongly agree shown as color coded where yellow depicting neutral while light blue as Agree and dark blue as Strongly agree).

**FIGURE 3:**
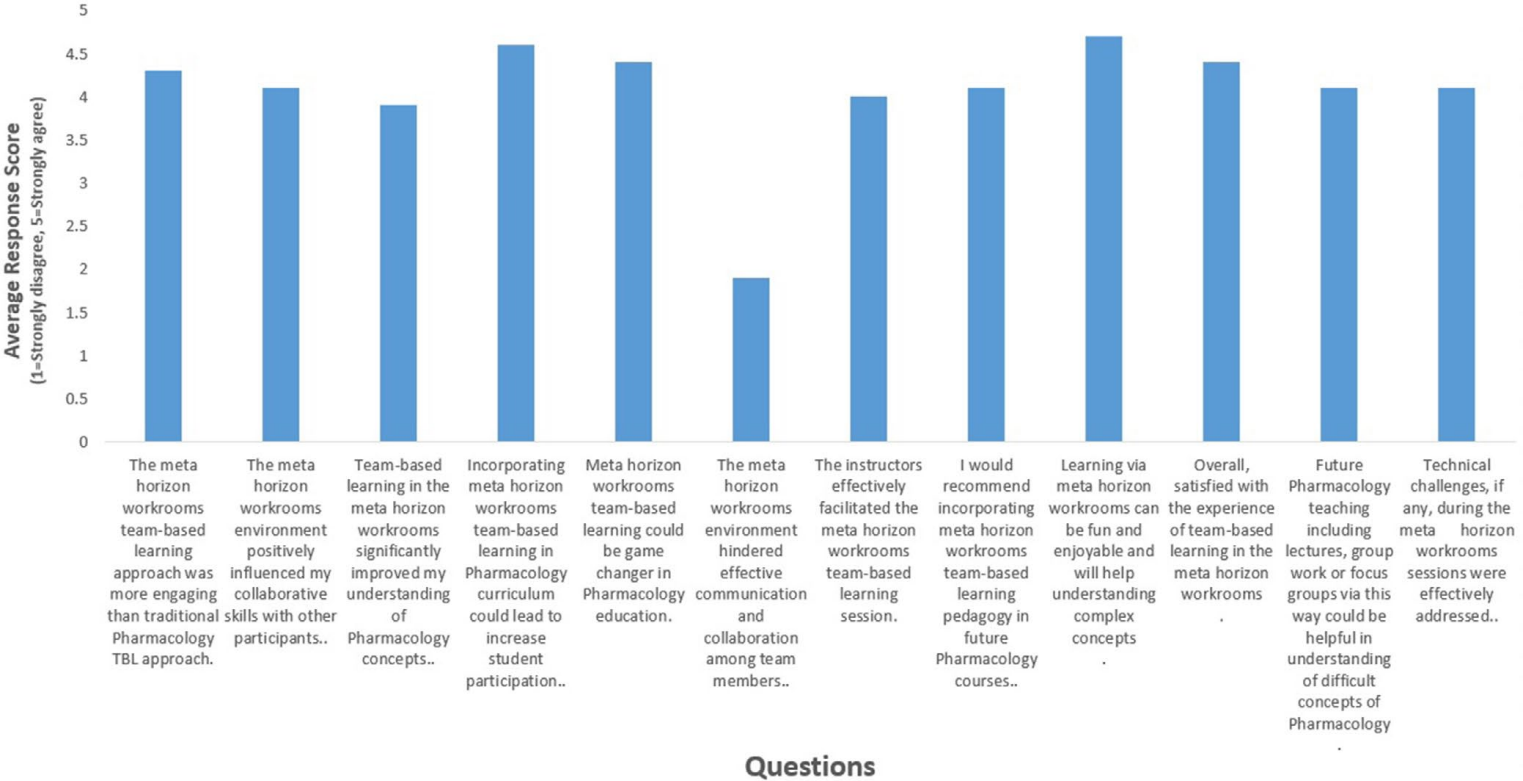
Average response analysis of Meta Horizon Workroom (MHW) team‐based learning (TBL) in pharmacology education. This graph illustrates the mean scores of student responses across various criteria, including engagement, collaboration, conceptual understanding, and overall satisfaction. The data collected using a 5‐point Likert scale (1 = Strongly disagree; 2 = Disagree; 3 = Neutral; 4 = Agree; 5 = Strongly agree) highlights the perceived effectiveness of the virtual environment in enhancing the learning experience.

Data collection involved both quantitative and qualitative methods. A 5‐point Likert scale questionnaire assessed students' perception of engagement, collaboration, and learning effectiveness. A fixed 5‐point Likert scale (1 = Strongly disagree, 2 = Disagree, 3 = Neutral, 4 = Agree, 5 = Strongly agree) was used, with no intermediate values. Averages for the Likert scales were calculated and plotted as a graph (Figure 3). Quantitative responses were analyzed using descriptive statistics and presented in the form of a figure (Figure 2). The open‐ended questionnaire provided qualitative insights into the strengths and challenges of the virtual TBL experience. Qualitative data was subjected to thematic analysis via NVivo version 14 [11], categorizing responses into themes: engagement, collaboration, motivation, and challenges. The thematic analysis process involved an inductive approach. Two independent researchers (A.K. and S.S.) read through all open‐ended responses to gain an initial understanding. They then discussed initial codes generated by NVivo software, which were then refined to develop broader themes. Any discrepancies in coding were resolved through discussion until a consensus was reached.

Teamwork and collaboration were primarily evaluated through self‐reported student perceptions via the Likert scale questionnaire, specifically statements addressing collaborative skills and communication within MHW. While direct comparative data to traditional TBL activities was not collected in this pilot, the student perceptions offer insights into the perceived improvement within the MHW environment. The open‐ended questions and initial codes used in this study are available as Data S1.

A total of eight students were recruited for this pilot study. This study's decision to only enroll 8 participants is supported by pragmatic factors resulting from resource limitations. The choice to maximize resources is influenced by the requirement for costly equipment and intensive training due to the MHW technology and TBL methodology. This reduced sample size manages the budgetary consequences of the expensive Oculus VR headset and participant training while enabling more effective use of staff and time. The decision is further supported by the limited availability of participants who possess pharmacological competence, which guarantees a targeted investigation of the intervention's effects in a reasonable and controllable way. This method, as a pilot study, fits with the exploratory character of determining viability, acceptability, and possible results prior to expanding the investigation.

## 3 Results

The results are based on data collected from 7 students as one student did not complete the questionnaire. The cybersickness questionnaire did not show any concern for any student pre and post MHW exposure.

### 3.1 Quantitative Data Findings

Five of the 7 students believed MHW‐based TBL was more engaging, enjoyable, and motivating to learn complex concepts compared to what they experienced in traditional TBL. Moreover, 6 of the 7 students found the VR environment interactive and collaborative and recommended using MHW in future pharmacology courses, while 4 of the 7 agreed (remaining 3 strongly agreed) that MHW could be a game changer in pharmacology education. Overall, 4 of the 7 students agreed, while the remaining 3 strongly agreed, that they were satisfied with their experience of using MHW for TBL (Figure 2). Students emphasized the ability to see and interact with peers in a shared virtual space as a key factor in their positive experience. The analysis of the Likert scale data reveals a mean score of approximately 4.00, suggesting that respondents generally provided positive or moderately high ratings (Figure 3). Technical issues, including audio distortions and problems logging in during the session, were reported by some participants.

### 3.2 Qualitative Data Findings

The following themes were generated.

#### 3.2.1 Theme: Engagement and Retention

Students described MHW TBL as highly engaging, attributing it to the immersive, distraction‐free environment. One student (S1) stated, “I feel the way we could engage more as a group means it will be a learning experience **in which** more information is retained due to the unique nature of the way the content is delivered and how we all collaborated to answer the questions.” Moreover, students reported better retention of pharmacology concepts. The interactive tools within MHW, such as the ability to display 3D models and collaborative whiteboards, supported visual learning and active engagement. One student (S2) noted, “The metaverse (MHW) is very immersive. It helps to engage students because of its interactive elements, like being able to manipulate virtual objects and write on shared whiteboards.”

#### 3.2.2 Theme: Collaboration and Communication

The ability to share digital resources in real‐time enhanced peer collaboration using VR was noted by a lot of the respondents as being one of the ways in which VR was positive. One student (S3) said, “Very useful to see people in the Meta Horizon Workroom and interaction made this more engaging and was easy to collaborate with shared documents and presentations.”

Other student (S4) said, “it boosted teamwork among peers and easy to communicate compared to classroom TBL.”

#### 3.2.3 Theme: Motivation and Enthusiasm

The novelty factor embedded in MHW acted as a leading force that boosted student motivation. Several students relayed that the virtual space works like a video game which brings both educational enjoyment and interest to learning. A student (S5) described, “It made the teaching more fun, engaging and stimulating. Due to the new experience the memory of teaching is more vivid and will stick in my mind for longer.”

#### 3.2.4 Theme: Technical Challenges

The system provided several advantages to students, but they faced minor usability problems such as echo, together with login processing difficulties. These issues, although minor, were mentioned by 2 participants and therefore warranted identification as a distinct theme reflecting barriers to a seamless user experience. A student (S6) said, “I struggled with echo and login which stopped me from fully engaged with the TBL however once its sorted out by faculty member then it was fine.” One student (S7) reported difficulty with VR interface initially but after provision of support the student engaged with the learning experience.

## 4 Discussion

The findings of this study align with previous research that highlights the advantages of VR‐based learning in medical education. Studies such as those by Lavoie et al. [12] and Parong and Mayer [3] have demonstrated that immersive environments can improve student engagement and knowledge retention compared to traditional teaching methods. In our study, 5 of the 7 students found MHW‐based TBL more engaging, reinforcing the fact that interactive and immersive platforms can enhance the learning experience. Moreover, collaboration and teamwork were positively influenced, a result that mirrors findings from research by Jensen and Konradsen [13], who reported that VR environments facilitate better peer‐to‐peer learning and communication. The ability to interact in a simulated clinical setting helps students develop critical thinking and problem‐solving skills, essential for medical education.

Our study aligns with research done by Radianti et al. [14], which found that the use of VR in higher education enhances knowledge retention by allowing students to engage with material more interactively. Students in our study highlighted the benefits of interaction, which contributed to a deeper understanding of pharmacology concepts. While direct measurement of knowledge retention was beyond the scope of this pilot study, our findings on enhanced engagement and deeper understanding suggest a mechanism through which retention could be improved, consistent with the literature. Additionally, the application exercise in this TBL session included knowledge‐based questions, and students' discussions during these exercises demonstrated improved recall and conceptual understanding of key pharmacological principles. This further supports the potential of VR to enhance retention through active learning. A broader review of medical education literature further supports these findings. For example, a systematic review by Kyaw et al. [9] on digital learning tools in medical education found that VR‐based methods were superior to traditional formats in improving both short‐ and long‐term retention of information. Similarly, Hamilton et al. [15] argued that VR‐based simulation training can enhance students' confidence and preparedness for real‐world clinical practice. Our findings, which indicate a higher level of engagement and participation in a virtual learning environment, further support these observations.

Another important aspect of our study was the perceived positive influence on collaboration through VR. Previous research by Dinca et al. [16] and Mcgrath et al. [17] using virtual reality simulation environments has shown that TBL in virtual environments fosters more equitable participation among learners, as VR settings reduce social anxieties associated with speaking in front of peers. This aligns with our findings, where students reported that their teamwork and communication within the virtual setting were positively influenced compared to traditional classroom‐based TBL.

Consistent with studies such as those by Kyaw et al. [9] our findings also reveal some technical challenges with VR technology. These challenges indicate that while VR‐based learning is highly effective, its integration into medical education requires further refinement to address technological barriers and improve user experience. According to Jensen and Konradsen [13], ensuring adequate training and user support is crucial in overcoming the initial learning curve associated with VR adoption. Our study reinforces this, as one student who struggled with VR echo and login disengaged in the learning experience initially. Audio distortions and problems logging in were the most reported issues. One student (S7) initially faced challenges with the VR interface, but after receiving support, they were able to engage effectively with the learning experience. Future VR applications in medical education may need to incorporate adjustable display settings and built‐in breaks to mitigate these issues.

Despite these challenges, the results of this study underscore the potential of MHW to transform pharmacology education by offering an engaging, interactive, and collaborative learning environment. By linking our findings with existing literature, we affirm that MHW‐based TBL can enhance engagement, teamwork, and knowledge acquisition, making it a valuable tool in modern medical education. The scalability of MHW for larger cohorts of students presents both opportunities and challenges. While the virtual environment can theoretically accommodate many users, practical considerations such as the availability of VR headsets, technical support infrastructure, and the logistics of managing numerous simultaneous sessions would need careful planning and investment. Future research would be required to develop strategies for scaling up this pedagogical approach effectively.

## 5 Limitations

Firstly, our sample size was relatively small, limiting the generalizability of the findings. This does not represent the whole cohort of the students having approximately 82 students. As mentioned before, a small sample was chosen for this pilot study due to resource limitations. A larger cohort of students would provide a more comprehensive evaluation of the effectiveness of MHW‐based TBL. Secondly, technical issues, including connectivity problems and occasional software glitches, posed challenges for a couple of students. Future research will explore ways to enhance the reliability and user‐friendliness of VR platforms. Additionally, our study focused primarily on student perceptions and engagement rather than objective learning outcomes. Future studies will incorporate pre and post‐tests to assess knowledge retention and skill development quantitatively. Longitudinal studies examining the long‐term impact of VR‐based learning on students' performance in clinical settings would also be beneficial. Also, the recruitment of students based on a pre‐existing level of “pharmacological competence” for this pilot study, while pragmatic for initial investigation, may limit the generalizability of the findings to the broader student population who may have varying levels of foundational knowledge. Future studies should consider recruiting a more diverse range of student competencies to assess the intervention's effectiveness across a wider spectrum of learners. Finally, the use of a convenience sample may have introduced selection bias, as students who were already interested or enthusiastic about VR‐based learning may have been more likely to volunteer to participate. This self‐selection could potentially skew the results towards more favorable perceptions and engagement levels. Future studies should aim to use randomized or stratified sampling methods to minimize this bias and ensure a more representative sample of the student population.

## 6 Conclusion

In conclusion, the integration of VR‐based learning environments represents a promising avenue in medical education, offering a valuable alternative to traditional TBL methods. The findings of this study, along with supporting research from the broader medical education literature, suggest that MHW could play a contributory role in the future of pharmacology education. Further research should focus on recruiting a large cohort of participants, refining VR‐based learning experiences, optimizing their usability, and addressing technical limitations to maximize their impact in educational learning.

## Author Contributions

**Abdullah Khaiyam:** conceptualization, data curation, formal analysis, investigation, resources, software, writing – original draft, writing – review and editing. **Lucy Battersby:** methodology, software, visualization, writing – review and editing. **Ellie Porter:** methodology, software, visualization, writing – review and editing. **Ana Correia de Oliveira:** methodology, resources, validation, writing – review and editing. **Soban Sadiq:** conceptualization, formal analysis, funding acquisition, investigation, project administration, resources, supervision, validation, writing – review and editing.

## Ethics Statement

This study was approved by KMMS REAG (Research Ethics advisory group) (Approval ID: 2405009) dated 20th September 2024.

## Conflicts of Interest

The authors declare no conflicts of interest.

## Supporting information


**Data S1:** prp270170‐sup‐0001‐DataS1.docx.

## Data Availability

The data that support the findings of this study are available from the corresponding author upon reasonable request.
