# Supplementary material for: Team‐Based Learning in the Meta Horizon Workroom: A Pilot Study on Its Potential Effectiveness for Pharmacology Teaching
Source: Pharmacol Res Perspect. 2025 Aug 25;13(5):e70170. doi: 10.1002/prp2.70170 (PMC12376176; doi:10.1002/prp2.70170)
Supplement: Supplementary file 1 — Data S1: prp270170‐sup‐0001‐DataS1.docx. [file PRP2-13-e70170-s001.docx]

**Supplementary data**

**Title: Team-based Learning in the Meta Horizon Workroom: A pilot study on its potential effectiveness for Pharmacology teaching**

Abdullah Khaiyam^1^, Lucy Battersby^1^, Elle Porter^1^, Ana Correia de Oliveira^1^ and Soban Sadiq^1^

^1^Kent and Medway Medical school, University of Kent, Canterbury, United Kingdom

Corresponding author: Soban Sadiq ([soban.sadiq@kmms.ac.uk](mailto:soban.sadiq@kmms.ac.uk))

**Likert scale questions**

1. The meta horizon workrooms team-based learning approach was more engaging than traditional Pharmacology TBL approach.
2. The meta horizon workrooms environment positively influenced my collaborative skills with other participants.
3. Team-based learning in the meta horizon workrooms significantly improved my understanding of Pharmacology concepts.
4. Incorporating meta horizon workrooms team-based learning in Pharmacology curriculum could lead to increase student participation.
5. Meta horizon workrooms team-based learning could be game changer in Pharmacology education.
6. The meta horizon workrooms environment hindered effective communication and collaboration among team members.
7. The instructors effectively facilitated the meta horizon workrooms team-based learning session.
8. I would recommend incorporating meta horizon workrooms team-based learning pedagogy in future Pharmacology courses.
9. Learning via meta horizon workrooms can be fun and enjoyable and will help understanding complex concepts.
10. Overall, satisfied with the experience of team-based learning in the meta horizon workrooms.
11. Future Pharmacology teaching including lectures, group work or focus groups via this way could be helpful in understanding of difficult concepts of Pharmacology.
12. Technical challenges, if any, during the meta horizon workrooms sessions were effectively addressed.

**Open-ended questions**

Question 1: Can you explain how immersive nature of meta horizon workrooms contributed to understanding and retention of pharmacology concepts compared to traditional methods?

Question 2: How team-based learning in the meta horizon workrooms facilitated peer-to-peer learning and collaboration in learning of topic discussed?

Question 3: In what ways did the meta horizon workrooms enhance your motivation and enthusiasm for learning Pharmacology concepts?

Question 4: What were the key challenges you faced using meta horizon workrooms environment?

Question 5: How would you describe your overall experience with team-based learning in the meta horizon workrooms for Pharmacology teaching?

**Thematic analysis Codes**

**Question 1: Understanding and retention**

**Codes:**

- Immersive environment:
- Interactive and collaborative learning
- Novel and engaging experience
- Practical features of VR

**Question 2: Peer-to-peer learning and collaboration**

**Codes:**

- Improved peer interaction
- Technical aids for collaboration
- Environment optimization

**Question 3: Motivation and enthusiasm**

**Codes:**

- Interactive and enjoyable learning:
- Novelty of VR
- Access to resources

**Question 4: Challenges faced**

**Codes:**

- Technical and ergonomic issues
- Audio difficulties
- Login issue

**Question 5: Overall experience**

**Codes:**

- Positive overall experience
- Potential for future integration
- Limitations in scope
